# Inter-epitope spacer variation within polytopic L2-based human papillomavirus antigens affects immunogenicity

**DOI:** 10.1038/s41541-024-00832-0

**Published:** 2024-02-24

**Authors:** Yueru Zhang, Filipe Colaco Mariz, Peter Sehr, Gloria Spagnoli, Karl Moritz Koenig, Simay Çelikyürekli, Tim Kreuziger, Xueer Zhao, Angelo Bolchi, Simone Ottonello, Martin Müller

**Affiliations:** 1 German Cancer Research Center, Im Neuenheimer Feld 242, 69120 Heidelberg, Germany; 2 EMBL-DKFZ Chemical Biology Core Facility, European Molecular Biology Laboratory, 69117 Heidelberg, Germany; 3 Department of Chemistry, Life Sciences and Environmental Sustainability, University of Parma, 43124 Parma, Italy; 4 Ruprecht-Karls University Heidelberg, Heidelberg, Germany

**Keywords:** Protein vaccines, Protein vaccines

## Abstract

The human papillomavirus minor capsid protein L2 is being extensively explored in pre-clinical studies as an attractive vaccine antigen capable of inducing broad-spectrum prophylactic antibody responses. Recently, we have developed two HPV vaccine antigens – PANHPVAX and CUT-PANHPVAX- both based on heptameric nanoparticle antigens displaying polytopes of the L2 major cross-neutralizing epitopes of eight mucosal and twelve cutaneous HPV types, respectively. Prompted by the variable neutralizing antibody responses against some of the HPV types targeted by the antigens observed in previous studies, here we investigated the influence on immunogenicity of six distinct glycine-proline spacers inserted upstream to a specific L2 epitope. We show that spacer variants differentially influence antigen immunogenicity in a mouse model, with the antigen constructs M8merV6 and C12merV6 displaying a superior ability in the induction of neutralizing antibodies as determined by pseudovirus-based neutralization assays (PBNAs). L2-peptide enzyme-linked immunosorbent assay (ELISA) assessments determined the total anti-L2 antibody level for each antigen variant, showing for the majority of sera a correlation with their repective neutralizing antibody level. Surface Plasmon Resonance revealed that L2 epitope-specific, neutralizing monoclonal antibodies (mAbs) display distinct avidities to different antigen spacer variants. Furthermore, mAb affinity toward individual spacer variants was well correlated with their neutralizing antibody induction capacity, indicating that the mAb affinity assay predicts L2-based antigen immunogenicity. These observations provide insights on the development and optimization of L2-based HPV vaccines.

## Introduction

Human papillomavirus (HPV) DNA was isolated from genital warts and cervical cancer biopsies by zur Hausen and colleagues in the late 1970s and early 1980s^1–3^. HPVs are designated as either high-risk or low-risk types, based on their potential to cause lesions, warts, or cancers^4,5^. High-risk mucosal HPV types are associated with the development of cervical cancer^6^. In the case of cutaneous HPV types, viral infection in combination with ultraviolet light DNA damage and cellular transformation in sun-exposed body sites has been linked to the development of non-melanoma skin cancer (NMSC) in immunosuppressed individuals^7^. Further, cutaneous HPV types are causing significant morbidity in organ transplant recipients, the majority of whom suffering from severe skin lesions within a few years after receiving the transplant.

Currently available HPV vaccines based on virus-like particles (VLPs) of the major capsid protein L1 have been proved to effectively prevent HPV infection and cervical lesions but these vaccines afford protection only against a subset of mucosal HPV types^8–10^. To achieve a broader breadth of protection and a more cost-effective production, in recent years, we have developed two vaccine candidates based on the minor capsid protein L2^11,12^. Our vaccine design is based on the hyper-thermostable thioredoxin (Trx) scaffold protein from *Pyrococcus furiosus*, in which a string of L2 epitopes (“polytope”) comprising the amino acids 20–38 region from different HPV types is inserted^13^. The presentation of repetitive L2 cross-neutralization polytopes within oligomeric nanoparticle structures is achieved by fusion of the OVX313 heptamerization domain to the C-terminus of the Trx scaffold^14,15^. The vaccine candidate targeting mucosal HPV types is designated as PANHPVAX, or Trx-L2m8mer-OVX313 in this work, and has recently entered a phase I safety and immunogenicity clinical trial (NCT05208710), whereas the vaccine candidate designated as CUT-PANHPVAX, or Trx-L2c12mer-OVX313, has been designed for the pan-targeting of cutaneous HPV types^12^. Previously, we showed that the L2-based antigens induce broadly protective immune responses in mouse and guinea pig models and afford in vivo protection against papillomavirus-induced infection and tumor formation in a naturally PV-sensitive rodent, *Mastomys coucha*^11,12,16^. Despite the broadly protective responses reported in the studies conducted with the Trx-L2m8mer-OVX313 and Trx-L2c12mer-OVX313 antigens, we observed a sub-optimal antibody-mediated neutralization of certain HPV types. For example, low neutralizing antibody titers were induced against HPV4 by the Trx-L2c12mer-OVX313 antigen and moderate neutralizing antibody titers against HPV31 by the Trx-L2m8mer-OVX313 antigens, respectively, notwithstanding the fact that both vaccine candidates harbor the corresponding L2 epitopes^11,12^.

Spacer or linker peptides are strings of amino acids that are inserted within the repeated portions of recombinant proteins in order to separate structurally defined domains while maintaining their function. In the case of repeated multi-epitope (“polytope”) antigens, spacer peptides were originally added to avoid so-called junctional epitope formation. Peptide spacers are empirically classified into three different categories: flexible, rigid, and cleavable linkers^17^. Glycine is widely used to form flexible linkers, as the small size of its side chain affords a high molecular flexibility^18^. Proline, with its cyclic side chain, favors a more rigid structure, and the lack of amide hydrogen precludes hydrogen bond formation with other amino acids, so that proline-rich spacers are quite effective in epitope separation^19^. In addition, an alpha-helix forming spacer is thought to behave as rigid due to hydrogen bonding-mediated stabilization^20^. As revealed by various studies, spacers with different flexibility differently affect protein folding, thus notwithstanding influencing different basic protein features, protein-protein interaction and biological function as well^21–25^. Spacer length also impacts on protein properties, and may interfere with the biological function of the fused protein^26^. In an analysis of a dataset of naturally occurring multi-domain proteins with known structure, it was observed that natural linkers comprise 10 ± 5.8 amino acid residues on average, with an average hydrophobicity index of 0.65 ± 0.09, and that longer spacer can cause an abnormally high solvent accessibility^18^.

Although the L2 amino terminus and its neutralization epitope is conserved even among distantly related papillomaviruses^27^, the exact structure and mode of presentation of the epitope within the viral capsid is not known, as it is the structure of the epitope bound to a neutralizing antibody. A medium size and flexibility spacer –the GGP tripeptide- was used in all our previous studies^11,12,15^. We thus asked whether changing the spacer at specific positions of our polytope vaccine candidates would impact, and possibly improve, antigen immunogenicity for certain HPV types. We also attempted to find out whether there is a generally beneficial type of spacer capable of improving antigen’s ability to induce neutralizing antibodies, or whether spacer effect varies with polytopes of different length and/or composition.

We thus set out to design and test spacers of different length and composition, i.e., made up by various combinations of glycine and proline capable of providing different degrees of flexibility. These were inserted at specific positions within the polytopes of the Trx-L2c12mer-OVX313 and Trx-L2m8mer-OVX313 antigens. The rationale for the choice of the upstream (aa 20) position for spacer substitution was based on our previous observation that the critical region for the induction of HPV-neutralizing antibodies is centered on the aa 20–31 region of the HPV-L2 aa 20-38 epitope^27^. We thus reasoned that the remaining portion of the epitope (aa 31–38), which is more conserved and likely involved in maintaining a proper structural conformation of the epitope, would serve by itself as a sufficiently extended spacer. Moreover, we were concerned that spacer modification/extension after aa residue 38 might have inadvertently affected the exposure of the subsequent epitope, which might have been properly exposed on its own, thus leading to a potentially confounding effect.

Each variant antigen was administered to mice biweekly for a total of four doses, and final sera from the immunized mice were collected for testing. The immunogenicity of antigen variants was evaluated further, and it was found to be positively correlated with the affinity of the antigens to a subset of HPV type-specific neutralizing monoclonal antibodies.

## Results

### Spacer variant incorporation into the HPV-L2 polytopes does not affect the biochemical properties of the antigens

**Fig. 1:**
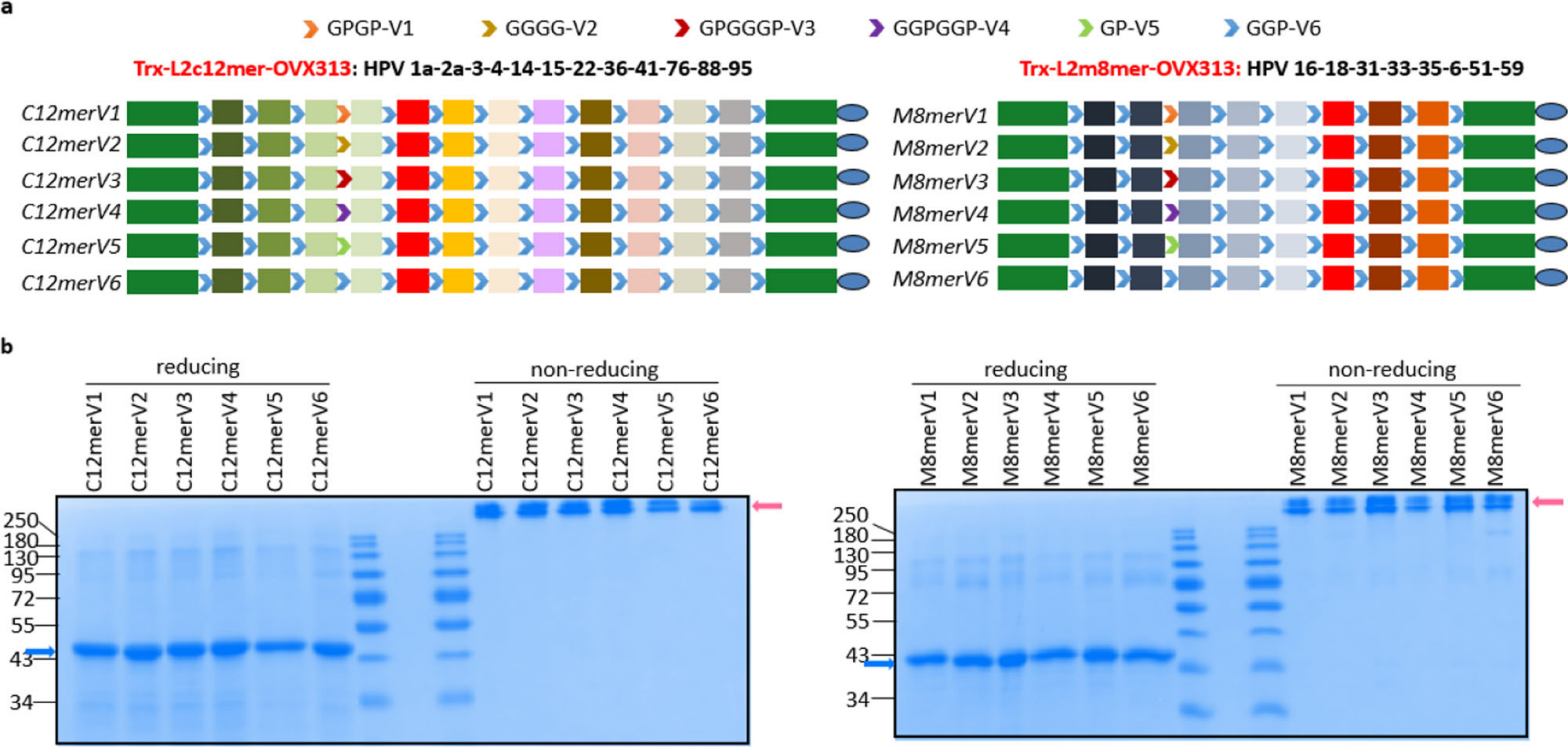
Recombinant expression, purification and stability/oligomerization properties of antigen variants harboring six different peptide spacers inserted into the L2 polytopes of cutaneous and mucosal HPV vaccine candidates. **a** Representation of the polytope and scaffold proteins compositions of Trx-L2c12mer-OVX313 (containing 12 different cutaneous HPV epitopes) and Trx-L2m8mer-OVX313 (containing 8 mucosal HPV epitopes). Six different spacers, composed of glycine (G) and proline (P) residues (indicated and color-coded at the top), were inserted between HPV3 and HPV4 L2 epitopes to generate antigen variants C12merV1 to V6, and between HPV18 and HPV31 L2 epitopes to generate antigen variants M8merV1 to V6, as indicated. The Trx and OVX313 scaffolds are indicated as green rectangles (shown on both sides of the polytopes) and as blue ovals, respectively. **b** All the proteins were efficiently purified and migrated as essentially uniform species (indicated by blue arrows) in SDS-PAGE under reducing conditions. Under non-reducing SDS-PAGE conditions, the heptameric, OVX313 disulfide-bonded forms of the antigens were preserved and migrated as larger size bands (marked by pink arrows).

A GGP tripeptide interposed between individual epitopes as well as at both ends of the display site of Trx (i.e., between the N- and C-terminal portions of the Trx scaffold and the first and the last epitope, respectively; see Fig. 1a) was previously used as a general spacer for the construction of our cutaneous (Trx-L2c12mer-OVX313^12^) and mucosal (Trx-L2m8mer-OVX313^11,15^) HPV-L2 vaccine prototypes. To explore the effect of localized spacer variations on the presentation and immunogenicity of specific epitopes, glycine and proline were assembled in different combinations to generate five other distinct spacer antigen variants. The five spacers and ‘GGP’, designated as V1-V6 (see Fig. 1a), were inserted between the HPV3 and the HPV4 L2 epitopes of Trx-L2c12mer-OVX313 (i.e., upstream to the epitope of the sub-optimally neutralized HPV4 type) and between the HPV18 and HPV31 L2 epitopes of Trx-L2m8mer-OVX313 (i.e., upstream to the epitope of the sub-optimally neutralized HPV31 type). In this way, six spacer variants for each antigen, designated as C12merV1 to C12merV6 and M8merV1 to M8merV6, were generated.

Following expression in Escherichia coli, a comprehensive characterization on antigen variants was conducted, involving solubility and thermal stability tests. All protein variants exhibited high solubility in lysis buffer and shared the same melting temperature, approximately 80 °C for C12mer variants and 75 °C for M8mer variants. The application of thermal treatment to the cleared lysate, followed by cation exchange chromatography, allowed similar purification protocols for all antigen variants. No differences in production yield, and purity level (above 90%) was observed among individual variants of the C12mer and the M8mer antigens, which displayed essentially identical subunit molecular weights upon SDS-PAGE analysis (46 kDa and 36 kDa for the C12mer and M8mer antigens, respectively).

Importantly, when analyzed under non-reducing SDS-PAGE conditions, all antigens migrated as high molecular weight species (marked with *pink arrows* in Fig. 1b), consistent with the formation of heptameric structures driven by the OVX313 multimerization domain present in all constructs.

### Spacer variation affects the neutralization immunogenicity of the corresponding antigens in a HPV type-related manner

**Fig. 2:**
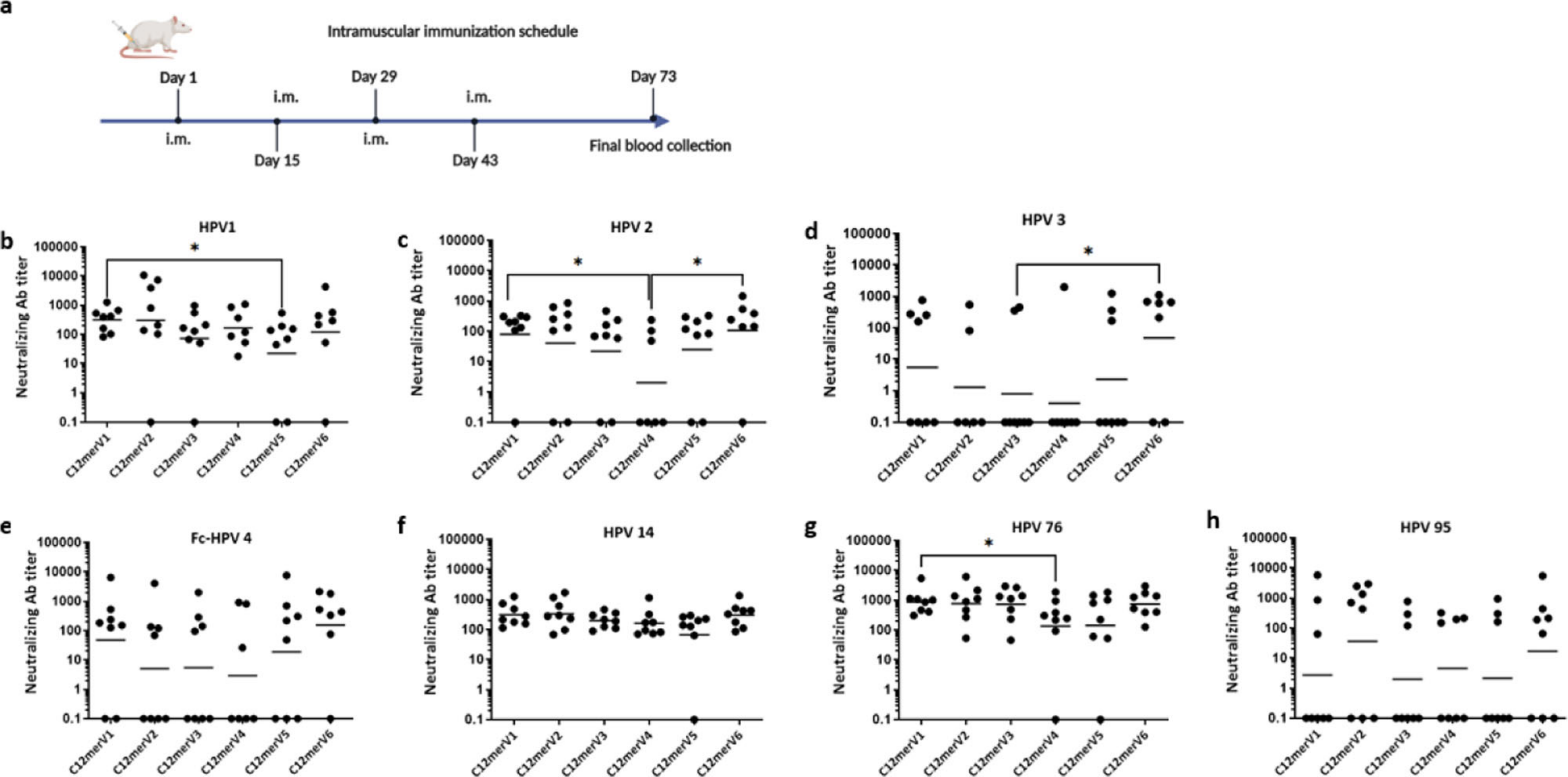
Antigen spacer variants differently affect the ability of Trx-L2c12merOVX313 to induce neutralizing antibodies against cutaneous HPV types in mice. **a** Groups of eight mice were immunized with different antigen spacer variants as indicated. **b**-**h** Neutralizing antibody levels against HPV1, HPV2, HPV3, HPV14, HPV76 and HPV95 were measured by PBNAs. Note that for HPV4 the higher sensitive Fc-PBNA was employed. Each data point represents the neutralizing antibody titer (EC50) measured in one mouse serum. Neutralizing antibody titers lower than 50 or EC50 with R square less than 0.85 were considered as non-neutralizing and set at 0.1. Horizontal bars represent the geometric mean titers (GMT) of each group. *P*-values ≤ 0.05, as determined by the nonparametric Mann–Whitney test, were considered significant and marked as ‘*’. **b** C12merV5 proved to be significantly less immunogenic than C12merV1 (*p*-value = 0.0482). **c** GMT of sera EC50 titers in the C12merV4 group was significantly lower than that measured in the C12merV1 and C12merV6 groups (*p*-values of 0.0263 and 0.0233, respectively). **d** Among the various weakly responding groups, anti-HPV3 neutralizing antibody levels in the C12merV3 was lower than those measured in the C12merV6 group significantly (*p*-value = 0.0493). **g** The ability of C12merV4 to induce neutralizing antibodies against HPV76 was significantly lower than that of C12merV1 (*p*-value = 0.0379). **e**, **f**, **h** No significant difference in anti-HPV4, anti-HPV14 and anti-HPV95 neutralizing antibody induction capacity was observed among the various antigen groups.

**Fig. 3:**
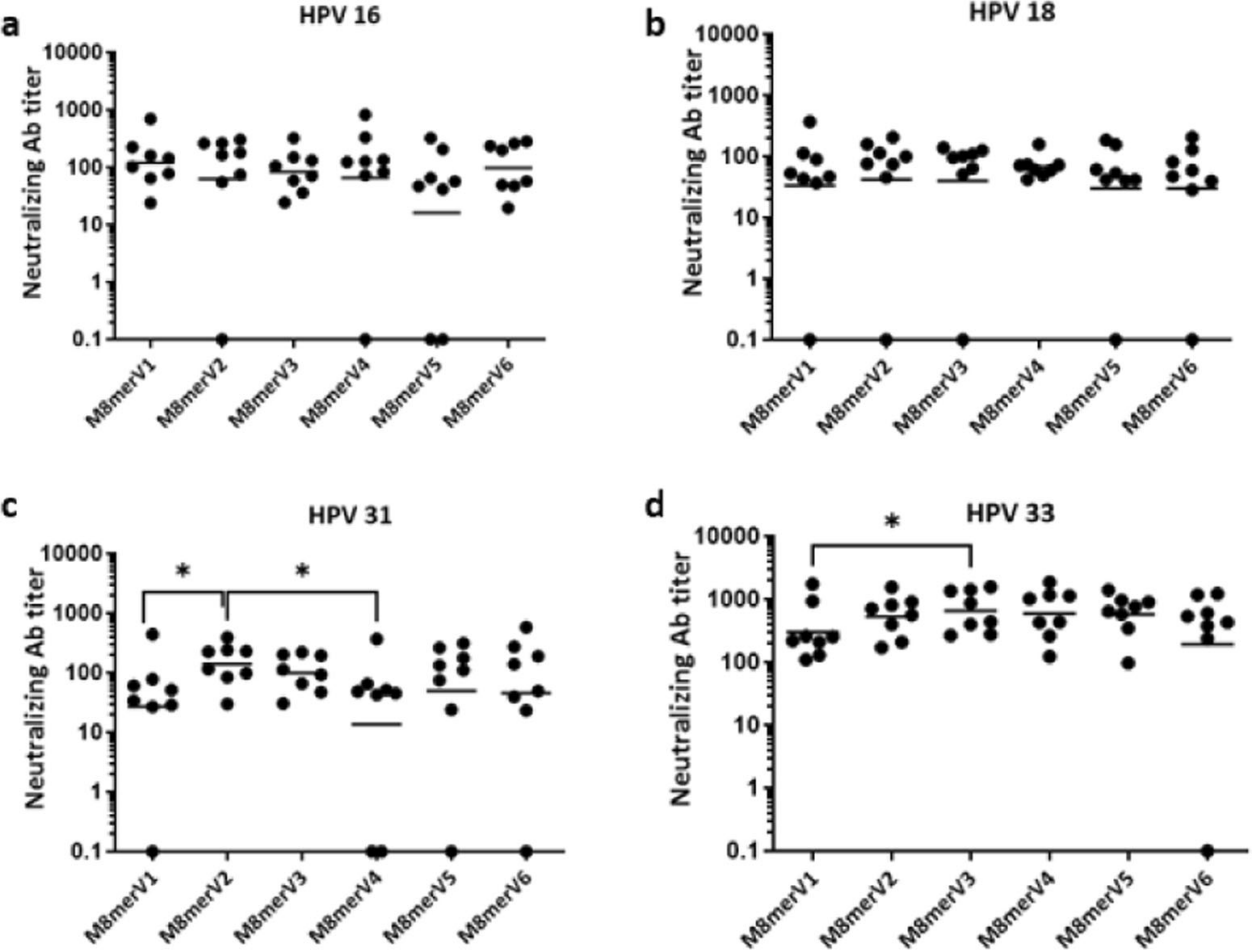
Effect of different spacer variants on the ability of Trx-L2m8mer-OVX313 to induce neutralizing antibodies against mucosal HPV types. **a**–**d** Neutralizing antibody levels against HPV16, HPV18, HPV31 and HPV33 induced by the indicated M8mer variants; data points represent neutralizing antibody titers (EC50) measured in individual mouse sera. Titers lower than 50 or EC50 with R square less than 0.85 were considered as non-neutralizing and set at 0.1. Horizontal bars represent the geometric mean titers (GMT) of each group. *P*-values ≤ 0.05, as determined by the nonparametric Mann–Whitney test, were considered significant and are marked as ‘*’. **a** Strong but not significant reduction of anti-HPV16 neutralization for the M8merV5 variant was observed. **b** Comparable anti-HPV18 neutralizing responses were induced in all antigen spacer variant groups. **c** M8merV2 significantly outperformed M8merV1 and M8merV4 in the induction of anti-HPV31 neutralizing antibody titers (p-value of 0.0379 and 0.0368). **d** M8merV3 induced significantly higher neutralization responses against HPV33 compared to the M8merV1 variant (*p*-value = 0.0499).

The purified antigens were formulated with the AddaVax^TM^ adjuvant, and four doses were injected intramuscularly into mice at two-week intervals (see Fig. 2a). Following blood collection one month after the last dose, the resulting sera were analyzed by pseudovirion-based neutralization assays (PBNAs). These were applied to a subset of the HPV types represented in the polytopes (seven out of 12 HPV types and four out of eight HPV types for the C12mer and the M8mer polytopes, respectively, including the sub-optimally neutralized HPV4 and HPV31 types; Figs. 2, 3). Our selection of the specific HPV types to be investigated, included those considered hard-to-neutralize (i.e., HPV4 for Trx-L2c12mer-OVX313 and HPV31 for Trx-L2m8mer-OVX313), as well as HPV types located upstream and downstream of the targeted epitope in the polytope string. All antigens induced detectable neutralizing antibody responses, albeit of varying strength, against the examined HPV types (Figs. 2, 3). None of the antigen variants, however, led to a generalized and consistently superior neutralizing antibody response against all the tested HPV types. Still, some statistically significant differences in the strength of the neutralizing responses elicited by some variant antigens against specific HPV types were observed. Most notable was the improvement of HPV1 and HPV2 neutralization associated with the variant antigen C12merV1 compared to C12merV5 and C12merV4 (p-values of 0.0482 and 0.0263, respectively), and the superior neutralization capacity against HPV2 and HPV3 displayed by the C12merV6 antigen compared to C12merV4 and C12merV3 (*p*-values of 0.0233 and 0.0493) (Fig. 2b–d). The C12merV1 variant also induced an anti-HPV76 neutralization response significantly stronger than that of C12merV4 (*p*-value of 0.0379) (Fig. 2g). No appreciable difference in neutralization capacity was observed with either variant against the other tested HPV types, including HPV4. Interestingly, the spacers contained in C12merV1 and C12merV6, the two antigens that elicited superior neutralization responses against the above reported HPV types, are four (GPGP) and three (GGP) amino acids in length, respectively, whereas shorter (two amino acids in the case of C12merV5) or longer (six amino acids for C12merV3 and C12merV4) spacers are associated to the more poorly performing antigen variants.

A different situation was observed in the case of the Trx-L2m8mer-OVX313 mucosal vaccine prototype, where a significantly superior HPV31 neutralization performance was found to be associated with the M8merV2 variant, which outperformed both the M8merV1 (*p*-value of 0.0379) and the M8merV4 (*p*-value of 0.0368) spacer variants. In the same context, M8merV3 elicited a superior neutralization response compared to M8merV1 against HPV33 (*p*-value of 0.0499), whose epitope is adjacent to that of HPV31 in the M8mer polytope. Interestingly, both best performing M8mer spacers (V2 and V3) differ from those we identified as best spacers in the case of the C12mer antigens (V1 and V6), as if the epitope spacing effect were somehow influenced by polytope length and/or composition. In addition, M8merV2 contains a highly flexible tetraglycine spacer, whereas a glycine-rich, six-residue spacer is present in M8merV3.

### Total and neutralizing anti-L2 antibody levels correlate among antigen spacer variants

**Fig. 4:**
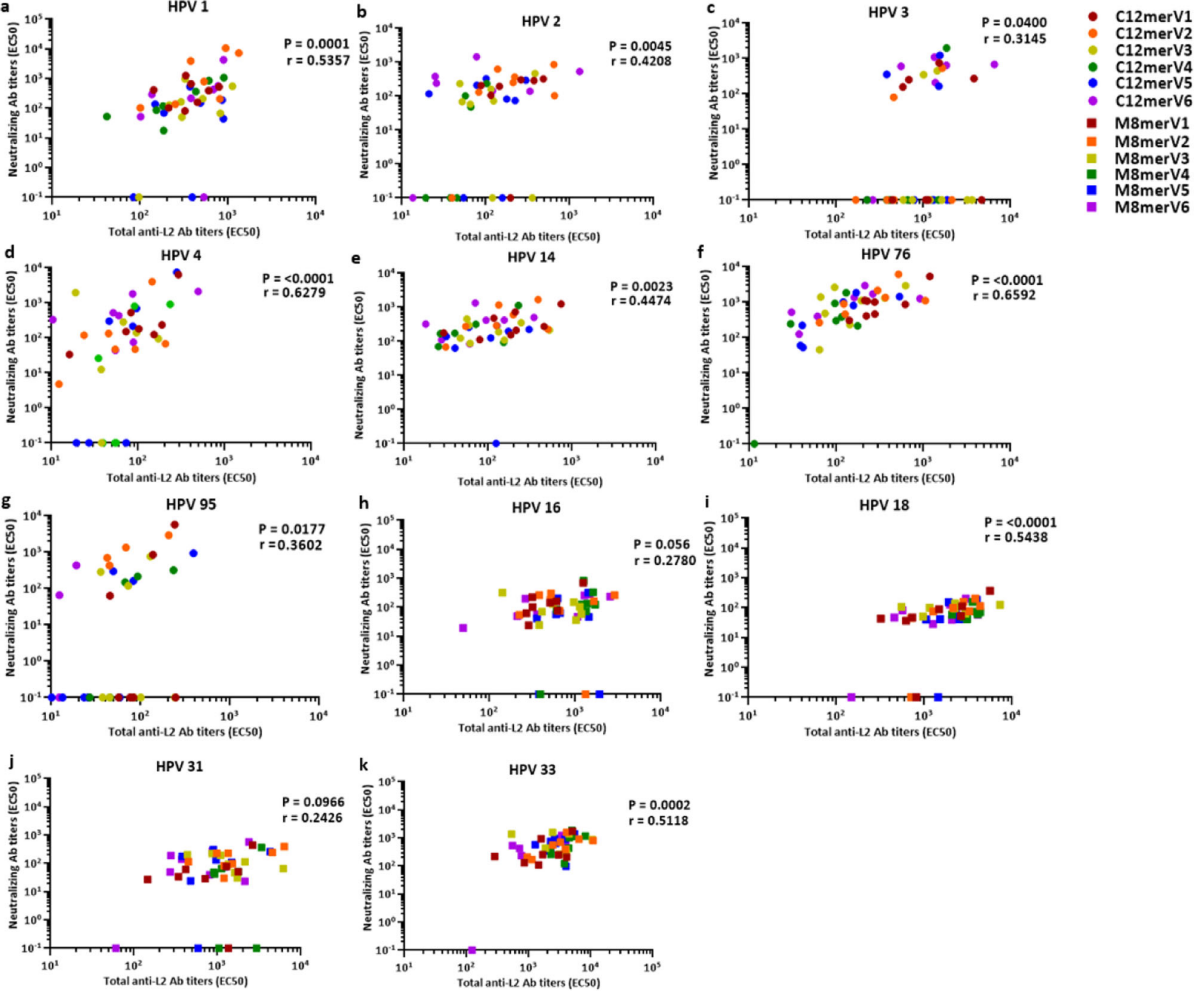
Neutralizing vs. total anti-L2 antibody titers induced by the different antigen spacer variants against distinct HPV types. **a**–**k** Neutralizing antibody titers (EC50, *y*-axis) determined by PBNA were correlated with total anti-L2 antibody titers (EC50, *x*-axis) measured by L2-peptide ELISA in individual immune-sera for cutaneous HPV types (HPV1, HPV2, HPV3, HPV4, HPV14, HPV76 and HPV95) and mucosal HPV types (HPV16, HPV18, HPV31 and HPV33). The shape and color of each data point coding for the different antigen groups are shown in the top right inset. EC50 values from PBNAs lower than 50 or EC50 with R square less than 0.85 were considered as non-neutralizing and set at 0.1. Similarly, EC50 values from ELISAs lower than 10 or EC50 values with R square less than 0.85 were considered as negative data-points and were excluded from analysis. Spearman’s rank correlation coefficients (r) for all mice sera (regardless of the antigen groups) and the corresponding p-values are provided inside each panel; the correlation was considered as statistically significant when the *p*-value was ≤0.05.

We then used peptide ELISA to determine the levels of total anti-L2 antibodies directed against the L2 epitopes of different HPV types. ‘Total anti-L2 antibodies’ refers to all the antibodies capable of binding to the targeted L2 epitope, irrespective of their neutralizing properties. The aim of this analysis was to explore the existence of a correlation between total and neutralizing anti-L2 antibody levels. In particular, we wished to determine whether a reduced neutralization capacity (or lack thereof) may correlate with a deficiency (or lack) of total anti-L2 antibodies against a certain epitope. Provided the effect of linker insertion on immunogenicity is more qualitative than quantitative, i.e. the antibodies induced are functional, then this should lead to a better correlation between peptide ELISA and PBNA. As shown in Fig. 4, ELISA data revealed measurable total anti-L2 antibody titers to each tested HPV-type L2 peptide for most sera, including sera with undetectable neutralizing antibody titers, such as those derived from mice immunized with the poorly performing C12merV3, C12merV4 and C12merV5 antigen variants (see Fig. 2).

Spearman’s correlation coefficients for total vs. neutralizing anti-L2 antibody titers were then calculated for a subset of cutaneous HPV types (seven out of twelve) and mucosal HPV types (four out of eight, see Fig. 4). For C12mer antigen variants, moderately strong (r > 0.5) positive correlations were found for HPV1, HPV4 and HPV76 (Fig. 4a, d, f, respectively), whereas weaker correlations were obtained for HPV2, HPV3, HPV14 and HPV95 (Fig. 4b, c, e, g, respectively). As to M8mer antigen variants, correlations for HPV18 and HPV33 were significantly strong (Fig. 4i, k, respectively), while no correlations were observed for HPV16 and HPV31 (Fig. 4h, j, respectively). Thus, for some but not all HPV types, total anti-L2 antibody levels strongly correlate with the neutralizing antibody titers induced by the various spacer variant antigens.

A more detailed representation (HPV vs. antigen variant type) of the correlation between L2-peptide ELISA titers and the EC50 values derived from the PBNAs for the corresponding HPV types is reported in Supplementary Table 1 and Supplementary Table 2. No significant correlation between total and neutralizing anti-L2 antibodies could be detected for any of the antigen variant groups for HPV3 and HPV14 for C12mer antigen variants, and HPV31 for M8mer antigen variants. In the case of HPV3, in particular, a large number of ELISA-positive immune-sera failed to display an appreciable neutralization capacity, and a similar lack of correlation was observed, conversely, for PBNA-positive sera. In respect to HPV14 and HPV31, most sera were neutralizing, yet ELISA and PBNA titers did not correlate. Looking at different antigen variants specifically, especially the V4-inserted variants showing comparatively poor performance in regard to immunogenicity (see Figs. 2, 3), we observed a significant correlation between total and neutralizing anti-L2 antibody titers for HPV1, HPV2 and HPV95 in C12merV4, and for HPV33 in M8merC4. For C12mer antigens, a fairly strong correlation was also observed for the antibody titers elicited by C12merV1 against HPV4, by C12merV2 against HPV1 and HPV95, and by C12merV5 against HPV4 as well as HPV76. In contrast, no significant correlation was observed with C12merV3 and the best-performing antigen C12merV6 for all the tested HPV types. In the context of M8mer antigens, a strong correlation was shown for the antibody titers induced by M8merV1 against HPV18, by M8merV2 against HPV18 and HPV33, and by M8merV6 against HPV16. Nevertheless, titers induced by the M8merV3 and M8merV5 antigens showed no significant correlation in respect to the two assays for all tested mucosal HPV types.

### Affinity of HPV type-specific monoclonal antibodies to different antigen variants is influenced by the inserted spacers and strongly correlates with immunogenicity

To gain further insight into the influence of inter-epitope spacers on HPV L2 polytope presentation, we set out to use Surface Plasmon Resonance (SPR) for a quantitative analysis of the interaction between all C12mer variants and a subset of neutralizing and non-neutralizing monoclonal antibodies (mAbs) targeting the L2 aa 20-38 epitopes of cutaneous HPV types 1, 2, 3, and 4^28^ (Supplementary Table 3). Since the heptameric, OVX313-containing antigens were stably bound to, and hardly dissociable from the mAbs (data not shown), for this analysis we employed the monomeric forms of the C12mer antigens. Non-reducing SDS-PAGE data showed that these OVX313-lacking, Trx-L2c12mer antigen derivatives were indeed fully monomeric and unable to oligomerize (Supplementary Fig. 1).

**Fig. 5:**
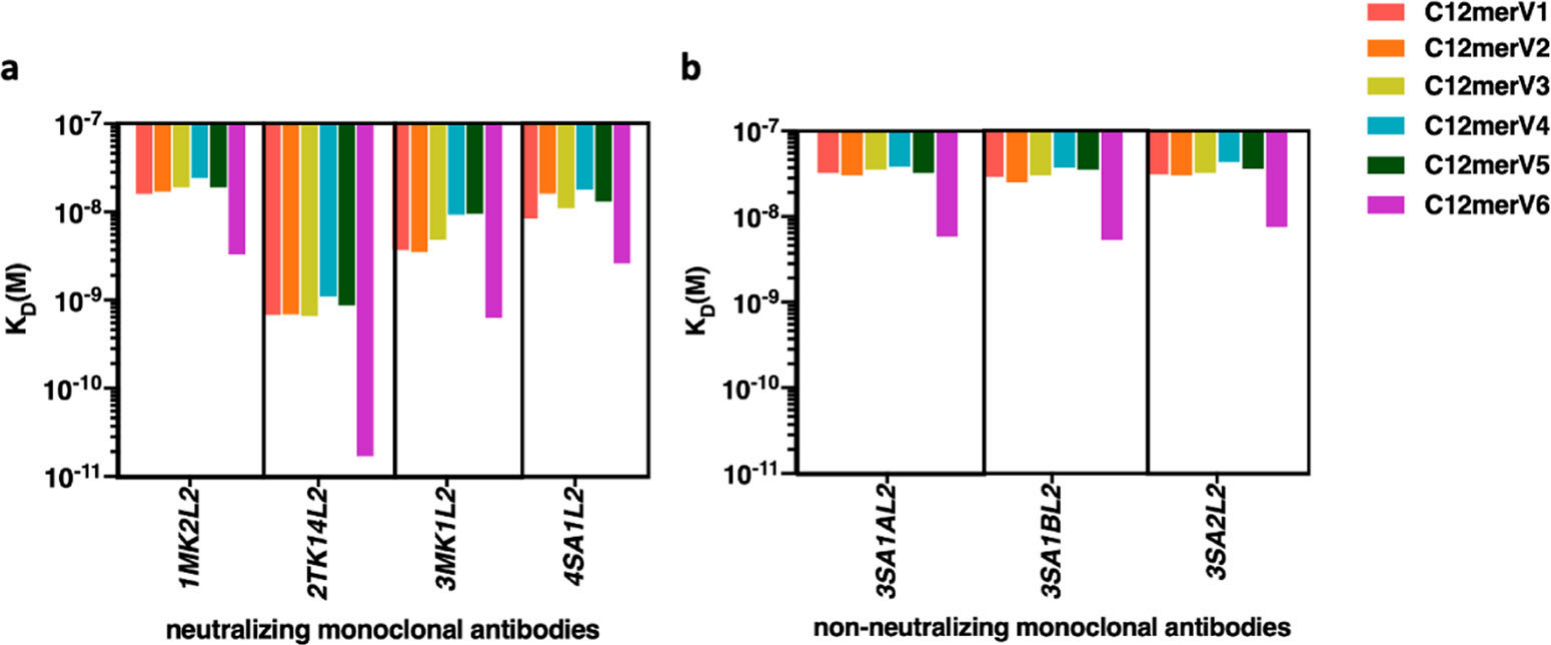
Variations in mAb affinity for antigen spacer variants arise from spacer modification. **a** Affinity of neutralizing mAbs 1MK2L2, 2TK14L2, 3MK1L2 and 4SA1L2 to six monomeric antigen variants (marked with different colors, as indicated) determined by SPR; K_D_ values are shown on the *y*-axis for different mAbs (*x*-axis) and antigen variants as indicated. **b** Same as (**a**) for the non-neutralizing 3SA1AL2, 3SA1BL2 and 3SA2L2 mAbs (see Supplementary Table 3 for the mAbs properties and target HPV specificities).

The affinity of four neutralizing (1MK2L2, 2TK14L2, 3MK1L2 and 4SA1L2, Fig. 5a) and three non-neutralizing (3SA1Al2, 3SA1BL2 and 3SA2L2, Fig. 5b) mAbs to the monomeric C12mer antigens was then determined based on the equilibrium dissociation constants (K_D_) provided by SPR measurements. The K_D_ values of non-neutralizing mAbs to all antigens (ranging from 5.3E-09 to 4.3E-08) were consistently higher than those of neutralizing mAbs (K_D_ values ranging from 1.7E-11 to 2.4E-08), indicating an overall lower affinity of non-neutralizing mAbs (Fig. 5).

**Fig. 6:**
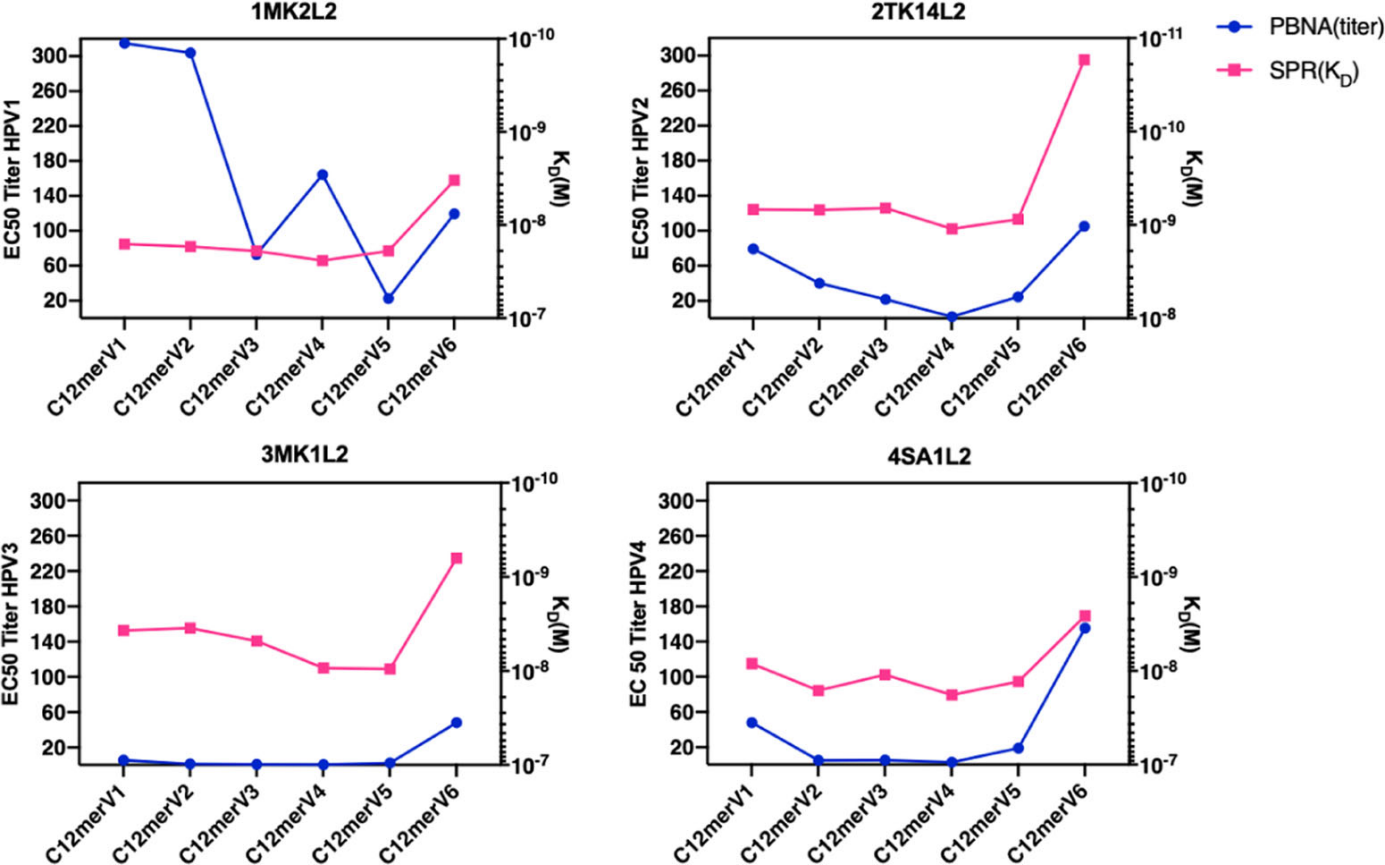
Correlation between the affinity of HPV type-specific neutralizing mAbs for different spacer variant antigens and the ability of such antigens to induce neutralizing antibody responses. In each graph, referred to different HPV types as indicated, the geometric mean of PBNA EC50 titers (left *y*-axis) are shown in *blue*, while the corresponding K_D_ values (right *y*-axis) determined by SPR for each monomeric antigen spacer variant (*x*-axis) are shown in *red*.

**Fig. 7:**
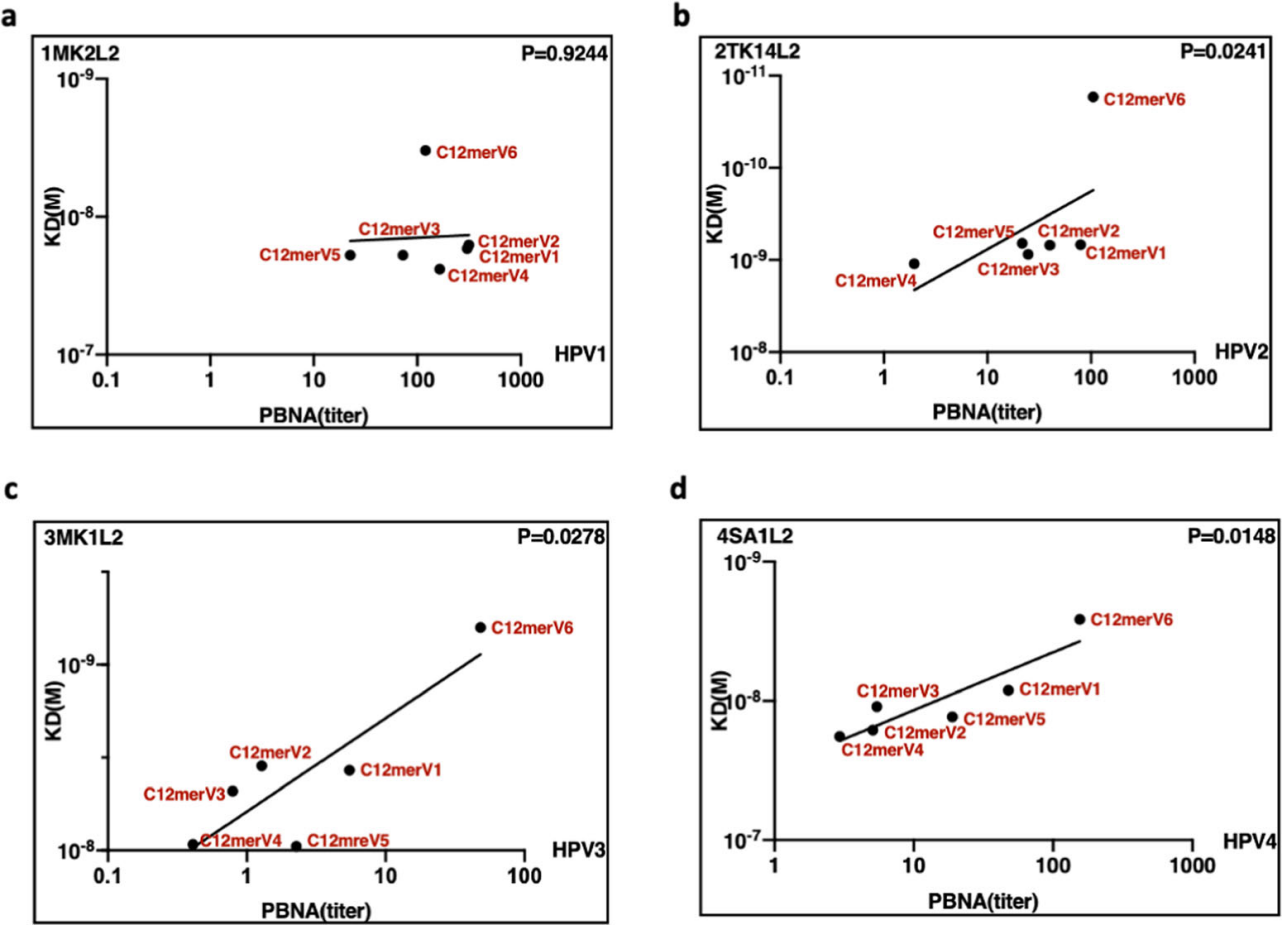
Linear correlation between K_D_ (SPR) and GMT (PBNA) values. **a**–**d** The dissociation equilibrium constants (K_D_, *y*-axis) from SPR tests were correlated with the GMTs of neutralizing antibody titers from the corresponding HPV-type PBNAs (*x*-axis). Each dot represents one antigen group as indicated. K_D_ (SPR) values for each monomeric antigen variant and mAb (indicated in each panel) are shown on the *y*-axis; GMT of EC50 (PBNA) values for the neutralization assays of the indicated HPV types are shown on the *x*-axis, respectively. *P*-values are reported within each panel under simple linear regression.

The monomeric form of the C12merV6 antigen, one of the best performing antigens revealed by cutaneous HPV neutralization assays (Fig. 2) displayed the highest affinity to all mAbs, with K_D_ values one-two orders of magnitude lower compared to the other variants. In contrast, C12merV4, one of the antigen variants with the weakest ability to induce neutralizing antibodies (Fig. 2), consistently exhibited the highest K_D_ values (i.e., lowest affinity) to all neutralizing mAbs. These results clearly indicate that spacer modification can influence mAbs affinity to the HPV1, HPV2, HPV3 and HPV4 L2 epitopes. In particular, as shown in Fig. 6, we found a consistent correlation between mAbs affinity to different spacer variant antigens and the geometric mean of the PBNA EC50 titers induced by such antigens. In fact, as further documented in Fig. 7, a linear relationship between SPR-derived K_D_ values and neutralizing geometric mean titers (GMT) was seen for HPV2-2TK14L2 (panel b; *p* = 0.024), HPV3-3MK1L2 (panel c; *p* = 0.0278) and HPV4-4SA1L2 (panel d; *p* = 0.0148).

Altogether, these data strongly suggests that K_D_ (i.e., mAb affinity) values measured by SPR may represent reliable predictors of neutralizing immunogenicity -much faster and more convenient to determine compared to the measurement of neutralizing antibody titers by PBNAs.

## Discussion

Current vaccines against human papillomavirus all rely on major capsid protein L1-based virus-like particles (VLPs), which have proven high efficacy and long-term protection^8–10^. The high multiplicity of the L1 immunogen on the surface of icosahedral VLPs promotes free trafficking through the lymph nodes and confers high immunogenicity to the VLP-based HPV vaccines^29,30^. However, their limited breadth of protection, complex manufacturing and storage requirements, including a strict cold-chain logistics, have prompted the development of new generation, more robust and broadly protecting HPV vaccines. Minor capsid protein L2 has been sought by various groups as the most promising alternative antigen because of its conservation across multiple HPV types and potential wide-ranging protection^27,31–33^. HPV infection is initiated by L1 binding to the heparan sulfate proteoglycans on the basement membrane, following by a conformational switch and an exposure of the amino-terminus of L2^34,35^. The L2 amino-terminus harbors a conserved furin cleavage site, that is located immediately upstream to the major cross-neutralization epitope^27,36,37^.

The two antigen candidates addressed in this paper comprise a string of L2 neutralizing epitopes from different HPV types inserted into a heptamerized thioredoxin scaffold. Both antigens (Trx-L2c12mer-OVX313 and Trx-L2m8mer-OVX313) induce neutralizing responses against a wide spectrum of cutaneous and mucosal HPV types, although antibody titers raised by the two L2-based recombinant antigens do not reach those induced by the exceptionally immunogenic VLP vaccines^11,12^.

In respect of vaccination, L1-VLPs display on their surface HPV type-specific neutralization epitopes arranged in a manner very similar or identical to that of native virions. In contrast, N-terminal L2-based neutralization epitopes are presented in a non-authentic context, also because VLPs containing both L1 and L2 do not elicit anti-L2 responses. The amino acid sequence of the L2 epitopes determines the basic secondary structure of the protein antigen. However, tertiary and quaternary structures are influenced by intra- and inter-molecular interactions established within the fully assembled L2 polytopic antigens. Initially introduced as a means to avoid the junctional epitope formation, inter-epitope spacers have subsequently been shown to impact the overall folding of subunit recombinant antigens^24,38,39^. In fact, successful priming of a B cell response depends not only on the exposure of individual neutralization epitopes, but also on the ‘native’ conformation of the displayed epitopes. In this context, ‘native’ means the acquisition of a three-dimensional structure that mimics as closely as possible the structure of the epitope within the natural virion. A three-dimensional (3D) structure of the L2 protein is not yet available and the use of structural prediction programs such as AlphaFold allowed to reliably model the overall 3D structure of the Trx-L2m8mer antigens but without enough resolution to pinpoint the structural variations associated to the introduction of different inter-epitope spacers (Supplementary Fig. 2). One major objective of this study is to determine experimentally if and how spacer variation influences epitope presentation in a qualitative manner, thus alters the antigen immunogenicity in respect to induction of neutralizing antibody responses.

Antigenic variants harboring the ‘GGPGGP’ spacer (C12merV4 and M8merV4) featured comparatively lower level of immunogenicity against the HPV types, which correspond to the HPV-type epitopes located close to the modified site. V4 has twice the length as the V6 (GGP), and such increased length may result in an enhanced flexibility of the juxtaposed epitopes. Insertion of the shortest length, ‘GP’ spacer (V5) also negatively affected immunogenicity compared to V6. C12merV5 displays weaker immunogenicity against HPV1, HPV3, HPV4, and HPV95, when compared to C12merV6; M8merV5 demonstrated lowest immunogenicity against HPV16 among all variants, albeit without a significant difference. The spacer with short length may stiffen the overall polytope, while increasing the interaction between adjacent epitopes and hindering the neutralization ability brought by the epitopes. In particular, as reported before, spacer length could influence the antigen ability to induce the production of high-affinity antibodies^26^. In light of the aforementioned findings, the three-residues-long GGP (V6) and the four-residues-long GPGP (V1) spacers appear to be the optimal ones for the cutaneous C12mer polytope. Instead, the flexible four-residues-long GGGG (V2) and, to some extent, the more rigid six-residues-long GPGGGP (V3) spacer maximize immunogenicity of the mucosal M8mer polytope. On the contrary, in view of the observed diminished immunogenicity of M8merV1 against HPV31 and HPV33, and the reduced immunogenicity of C12merV3 against HPV3, it is reasonable to consider that the spacer effect is also related to the overall length of the polytope.

One important limitation to be addressed in our investigation of antigen immunogenicity influenced by spacer modification is that, in order to sensitively recognize the differences among closely related groups, a larger sample size would be required. However, this size is already sufficient to yield a statistically significant *p*-value (less than 0.05) if there is a relevant difference present in the data. For example, the two-sided Mann–Whitney test used here will have a statistical power of 80% already at *n* = 8, if the effect size is 1.6 standard deviations or more. Additionally, it should be noted that the immunogenicity titers elicited by C12merV6 and M8merV6, in comparison to the historical titers obtained previously, generally exhibit consistency but not align perfectly. This variation can be attributed to several factors that can influence the precise titer levels, including discrepancies in antigen purity, variations in the mouse batches used, and the use of distinct PSV preparations in PBNA. While minimizing procedural variability is a priority, a certain degree of titer difference is considered acceptable in different PBNA experiments.

HPV L2 peptide ELISA was used to determine total anti-L2 antibody titers, regardless of their neutralization ability. This measurement lacks specificity and may also detect the cross-reactive antibodies, due to the highly conserved L2 epitope sequences among different HPV types. The correlation between neutralizing antibody and total anti-L2 antibody titers was observed for all cutaneous HPV types and half of the tested mucosal HPV types, suggesting an optimal display of the epitopes for these particular HPV types within the recombinant antigens. However, the correlation is quite moderate overall, which could be attributed by the lower specificity of the ELISA and the lower sensitivity provided by the standard PBNA. Such correlation was not consistently maintained when the analysis was performed within distinct antigen variant. This may be due to a sample size reduction, which may amplify the variation in total anti-L2 antibodies resulting from the cumulative detection of cross-reactive and non-neutralizing antibodies by ELISA. Nevertheless, significant correlations were consistently observed for some antigen groups, such as C12merV2, C12merV4, C12merV5 and M8merV2. In this case, total antibodies detected by L2 peptide ELISA can serve as a surrogate, easier to measure indicator of the levels of neutralizing antibodies against the corresponding HPV type. However, additionally, more detailed investigations will be required in order to fully interpret these findings and translate them into an improved (more effective) vaccine design methodology.

Furthermore, we also used SPR to investigate the influence of different spacers on the affinity of type-specific mAbs for the corresponding monomeric antigen variants. There, we found an overall significant positive correlation between the immunogenicity (i.e., neutralization capacity) of different antigen spacer variants and their affinity for neutralizing mAbs. For example, C12mer antigen variant harboring the ‘GGP’ spacer (V6) displayed high affinity for the neutralizing mAbs directed against HPV1, HPV2, HPV3, and HPV4 L2 epitopes as well as a high neutralization capacity for the corresponding pseudovirions. This emphasis lies on the correlation between the in vitro antigen affinities to mAbs and the in vivo antigen immunogenicity. Given the in vivo immunogenicity evaluation is laborious and time consuming, SPR determination of antigen variant affinity for neutralizing mAbs lends itself as a valuable alternative in vitro methodology for pilot-scale evaluation and ranking of new candidate antigens.

Neutralization is the process in which the viral productive infection is inhibited through antibody binding of viral particles. In contrast, non-neutralizing antibodies can interact with viral immunogens, whereas the infectivity of the virus is retained. The non-neutralizing mAbs, namely 3SA1AL2, 3SA1BL2 and 3SA2L2, were generated from mice immunized with HPV3 L2 neutralizing epitope. It would be conceivable to imagine that incorrect or otherwise sub-optimal epitope might be revealed by assessment of antigen affinity to non-neutralizing mAbs. Nevertheless, the affinity pattern of six antigen variants for both neutralizing and non-neutralizing mAbs remains consistent, with C12merV6 displaying the highest affinity and C12merV4 the lowest affinity for both. Despite this, it is worth noting that the affinity observed between antigen variants and neutralizing mAb_3MK1L2 are consistently 10- to 30-fold higher than the affinity seen with non-neutralizing mAbs. This implies that the failure of non-neutralizing mAbs is caused by the low binding strength to the antigen immunogen^40^.

In conclusion, this study highlights the impact of spacer selection for an antigenic protein in regard to functional immunogenicity, especially polytopic antigens, and points to medium length spacers as the best initial choice. It also documents the effectiveness of SPR-assisted determination of candidate antigen affinity for type-specific mAbs as a reliable in vitro preliminary tool of more laborious in vivo immunogenicity assays. However, the acquisition of comprehensive information on antigen-induced antibody titers, persistence and other related factors requires in-depth examination through in vivo studies on those selected antigen candidates.

Furthermore, our comprehensive analysis of antigen variant immunogenicity comparing to the reference spacer V6 antigen (PANHPVAX or CUT-PANHPVAX) suggests that no necessary adjustment to the spacer is warranted in the ongoing and coming clinical trial. Still, our study addresses the importance of spacer selection in enhancing antigen efficacy and immunogenicity. The spacer effect on antigen remains a critical consideration in the methodological design of optimal multi-domain protein antigens. This knowledge contributes to the broader understanding of protein antigen engineer and will continue to guide future strategies for the optimization of multi-domain vaccines.

## Methods

### Expression and purification of proteins

Synthesized DNA plasmids encoding the target proteins were transformed into *E.coli*. Protein expression was induced at optimal concentrations of Isopropyl β-D-1-thiogalactopyranoside (IPTG), ranging from 0.5 and 2 mM IPTG. Induction was performed at room temperature overnight under 100 rpm shaking conditions. Cells were lysed in 300 mM NaCl, 25 mM Tris-HCl, 0.16% Tween-20, 1 mM phenylmethylsulfonyl fluoride (PMSF) and 0.1 mg/ml lysozyme at pH 8 using a French press. The cleared lysate was heated at 75 °C for 30 min followed by centrifugation to remove denatured host cell proteins. The antigens were further purified by cation-exchange or affinity chromatography. To this end, protein samples were equilibrated at pH 8 or pH 7.5, for C12merV6 and C12merV1-V5 as well as M8merV1-V6, respectively, and then loaded onto a HiTrap Sepharose Fast Flow column (C12merV6 and M8merV1-V6) or a Heparin affinity column (C12merV1-V5). Elution was performed by linear salt gradients and antigen containing fractions, detected by SDS-PAGE, were pooled and exchanged into 1 X PBS buffer using Amicon® Ultra-15 Centrifugal Filters. Endotoxin was removed with 1% (v / v) Triton X-114^41^. Final antigen concentration was determined by SDS-PAGE, in comparing with a protein-standard of bovine serum albumin (BSA). Gels from the same experiment were processed in parallel.

### Animal permit

BALB/c mice were kept in compliance with German and European statutes at the German Cancer Research Center and all animal experiments were carried out with the approval of the responsible Animal Ethics Committee: Regional Council of Karlsruhe, Germany; 35–9185.81/G-248/16.

### Mouse immunization

Six to Eight week-old BALB/c female mice were purchased from Charles River Laboratories (Sulzfeld, Germany) and were kept under specific-pathogen-free conditions. 1 X PBS buffer was used to dilute the antigens to reach the 20 µg / 50 µl dose per mouse. 50% (v / v) AddaVax^TM^ (InvivoGen) was used as adjuvant and added to the diluted antigen. AddaVax is an oil-in-water nanoparticle emulsion containing squalene, and shares similarities with MF59^42^. The antigen thus formulated was injected intramuscularly into each mouse for a total of four immunizations, separated by two-week intervals. The intramuscular injection was administered into *m. tibialis anterior*. Animals were sacrificed using CO_2_ chamber one month after the last dose and final blood samples were collected.

### Pseudovirion (PSV) preparation

The human fibroblast cell line 293TT transfected with plasmids carrying humanized HPV L1 and L2 coding sequences plus a reporter plasmid was used for the preparation of PSVs, which were then purified by Optiprep step gradient ultracentrifugation as described previously^11,43,44^.

### Pseudovirion-based neutralization assay (PBNA)

The pseudovirion-based neutralization assay (PBNA) was used to detect anti-HPV neutralizing antibodies. Briefly, 50 µl of immune-sera diluted in Dulbecco modified Eagle medium [DMEM] were combined with 50-µl of DMEM pre-diluted pseudovirion and incubated at room temperature for 20 min. Next, 50 µl of HeLa T cells (2.5 × 10^5^ cells/ml) were added to the pseudovirion antibody mixture and incubated for 48 h at 37 °C in a humidified incubator. The amount of secreted Gaussia luciferase was determined in 10 µl of cell culture medium using the Gaussia Glow Juice kit according to the manufacturer’s instructions (PJK GmbH, Germany). Sample luminescence was measured 15 min after substrate addition.

Fc-PBNA was performed with furin-cleaved PSV produced as above except using a furin producing cell line (293TT-F) for production and LoVoT cells for transduction.

### HPV L2 peptide ELISA

The L2 peptides were synthesized as N-terminally conjugated biotin-containing derivatives of the HPV1, HPV2, HPV3, HPV4, HPV14, HPV76, HPV95 L2, HPV16, HPV18, HPV31 and HPV33 epitopes. In total, we performed eleven different HPV L2 peptide ELISAs. A polyvinyl chloride, 96-well plate was coated overnight with streptavidin (1 mg/ml in stock, 1:300 diluted in sterile MQ water) at 4 °C. After blocking with 1.5% (w/v) milk, the tested biotinylated L2 peptide was diluted 1:400 in blocking-buffer (1.5% milk) and added to the coated plate. After a 1-hour incubation at room temperature, the plate was washed three times with 0.3% PBST (0.3% (v / v) Tween-20 in 1 X PBS). Then, different dilutions of individual serum in 1.5% (v / v) milk were added to the wells, followed by a 1-hour incubation at 37 °C and three additional washings with 0.3% PBST. A secondary, horseradish peroxidase (HRP)-conjugated goat anti-mouse IgG (Dianova - 87584) diluted 1:3000 in 1.5% milk, was then added to the plate and incubated for 1-hour incubation at 37 °C. After a further washing with 0.3% PBST, the plate was developed by 2-2’-azino-di-(3-ethylbenzthiazoline sulfonic acid) (ABTS) plus of H_2_O_2_. The plate was then read 5–10 min after substrate addition. EC50 values were derived from sera dilutions yielding half-saturated signals.

### Surface plasmon resonance assay

SPR measurements were performed with a BIACORE T200 (Cytiva) and PBS containing 0.02% Tween20 and 0.05% BSA as running buffer. An anti-mouse IgG antibody sensor chip was prepared by covalent immobilization of rabbit anti-mouse IgG antibodies (Mouse Antibody Capture Kit, Cytiva) to a SCBS HC30M-chip (Xantec) as descripted in the kit.

Antibody concentration in hybridomas was analyzed with this sensor chip using mouse immunoglobulin as standard to adjust a similar capture level (50 RU) of antibodies (ligand) in all further binding experiments. Naïve mouse IgG captured to a second flow cell served as reference.

A fivefold 1:4 serial dilution of the C12mer antigens (analyte) starting at 1000 nM was injected in sequence in a single cycle and an additional cycle with buffer injection was applied for double referencing. The chip surface was regenerated between cycles with glycine-HCl pH 1.7 supplied with the mouse antibody capture Kit. Sensorgrams were fitted with a kinetic 1:1 model in the Biacore software and the equilibrium dissociation constant K_D_ was calculated.

### Structure prediction

The structures of the GGP-containing variants (V6) of the Trx-L2m8mer and Trx-L2c12mer antigens were first predicted with AlphaFold 2, which yielded a reliable prediction for the PfTrx scaffold (PIDDT score ranging from confident to very high) but not for the 8mer or 12 mer polytope (PIDDT score: very low) portions of the antigens. The polytope regions were thus modelled with the Modeller program, using as reference the structure of the N-terminal, aa 20-38 region of L2, extracted from the structure of full-length L2 predicted with AlphaFold 2 (PIDDT score of the L2 aa 20–38 region: confident to very high). The structures of the other spacer variants (V1-V5) were then predicted with the SWISS-MODEL server, using as reference the structure of the GGP-containing variants; the Chimera program was used for overlaying the six structures.

### Statistical analysis

GraphPad Prism 8.3.1 was used to calculate EC50 from PBNA and EC50 from peptide ELISA, as well as the geometric mean of mouse-sera titers from each antigen group. The significant difference of two groups was determined with the nonparametric Mann-Whitney test, also using GraphPad. When *p* value is ≤0.05, the difference is considered as statistically significant. In addition, the correlation between PBNA and ELISA was determined using Spearman correlation by GraphPad. A *p* value ≤ 0.05 was considered a significant correlation.

### Reporting summary

Further information on research design is available in the Nature Research Reporting Summary linked to this article.

### Supplementary information

Supplemantary information

REPORTING SUMMARY

## Data Availability

All the important data and figures were shown in this manuscript. Additional detailed data can be reasonably requested by contacting the first author or the corresponding author.
