# Supplementary material for: Inter-epitope spacer variation within polytopic L2-based human papillomavirus antigens affects immunogenicity
Source: NPJ Vaccines. 2024 Feb 24;9:44. doi: 10.1038/s41541-024-00832-0 (PMC10894200; doi:10.1038/s41541-024-00832-0)
Supplement: Supplementary file 1 — Supplemantary information [file 41541_2024_832_MOESM1_ESM.pdf]

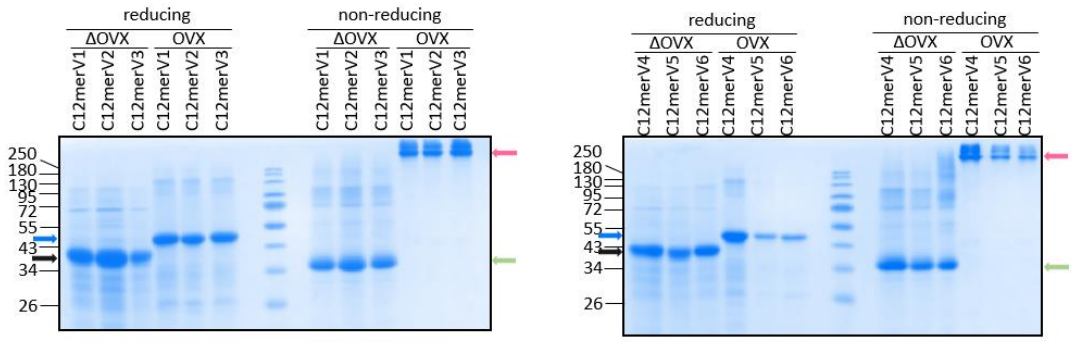

Supplementary Figure 1. Protein variants lacking the OVX domain exhibit a monomeric structural conformation. C12merV1-V6 marked 'ΔOVX' represent the protein variants lacking the OVX domain, which were used in the affinity tests with mAbs. The pairwise comparison of the protein variants, lacking or harboring the OVX domain via SDS-PAGE under reducing and non-reducing condition, reveals the loss of oligomerization resulting from the removal of the OVX domain. The arrows indicate the localization of the different proteins.

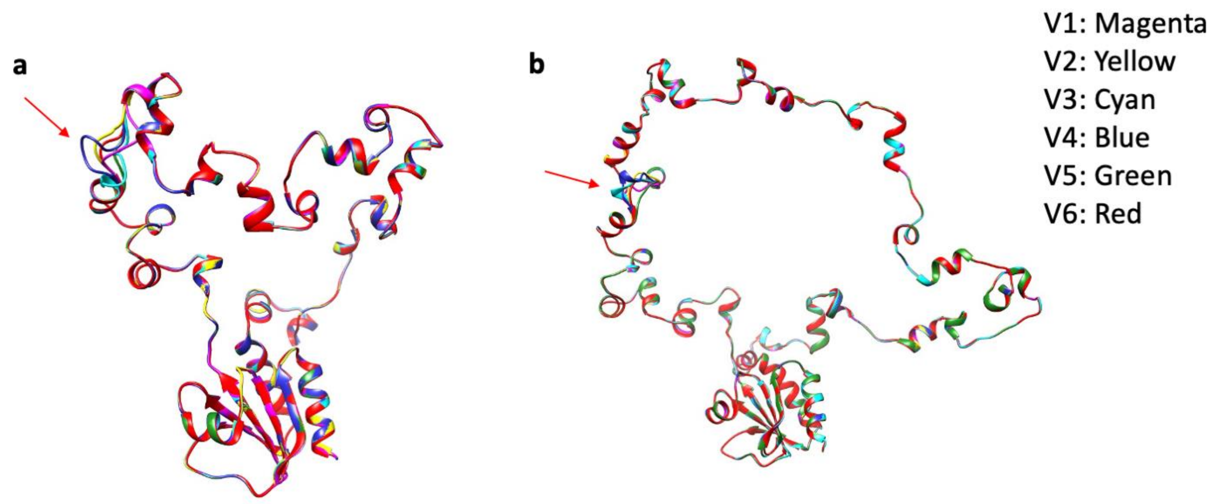

Supplementary Figure 2. (a) Overlaid predicted structures of the mucosal HPV, Trx-L2m8mer spacer variants (monomeric form w/o OVX313). The color codes for the different spacers, interposed between the HPV18 and the HPV31 epitopes (see Figure 1a), are indicated in the inset; the GGP spacer (V6, *red*) is present at all the other inter-epitope positions. The predicted structure of the GGP spacer-containing variant was used as a reference, on which the predicted structures of all the other variants were overlaid; the position of the variant spacers in the overlaid predicted structures is marked by a *red* arrow (see 'Materials and Methods' for details on structure prediction). (b) Same as (a) for the cutaneous HPV, Trx-L2c12mer spacer variants. The different spacer variants were inserted between the HPV3 and HPV4 epitopes (insertion position marked by a *red* arrow; see also Figure 1a); the GGP spacer (V6, *red*) is present at all the other inter-epitope positions.

Supplementary Table 1. Spearman correlation analysis of the relationship between total (L2-peptide ELISA) and neutralizing (PBNA) antibody levels, listed according to each C12mer antigen group and HPV type.

HPV type	Spearman Analysis	C12mer V1	C12mer V2	C12mer V3	C12mer V4	C12mer V5	C12mer V6
	p value	0.5364	0.0341	0.1710	0.048	0.564	0.0663
**HPV1**	Spearman r	0.2619	0.8214	0.5476	0.7857	0.2395	0.75
	p value summary	ns	*	ns	*	ns	ns
	p value	0.115	0.4444	0.9159	0.0286	0.8063	0.556
**HPV2**	Spearman r	0.619	0.3571	-0.0479	0.8669	0.1078	0.2857
	p value summary	ns	ns	ns	*	ns	ns
	p value	0.631	0.6667	0.8214	0.3333	0.875	0.0897
**HPV3**	Spearman r	0.2029	0.2704	-0.0935	0.6547	0.0818	0.7027
	p value summary	ns	ns	ns	ns	ns	ns
	p value	0.0368	0.4976	0.6135	0.25	0.0381	0.3956
**HPV4**	Spearman r	0.7619	0.3214	0.2342	0.5768	0.8154	0.3929
	p value summary	*	ns	ns	ns	*	ns
	p value	0.0962	0.3536	>0.9999	0.175	0.3894	0.1389
**HPV14**	Spearman r	0.6429	0.4286	0.00	0.6571	0.3571	0.6429
	p value summary	ns	ns	ns	ns	ns	ns

**HPV76**	p value	0.2992	0.0881	0.2162	0.4444	0.0154	0.1389
	Spearman r	0.4286	0.7143	0.5	0.3571	0.8333	0.6429
	p value summary	ns	ns	ns	ns	*	ns
**HPV95**	p value	0.619	0.0071	0.875	0.0381	0.0536	0.6429
	Spearman r	0.2182	0.9349	-0.0818	0.8078	0.7365	-0.2224
	p value summary	ns	**	ns	*	ns	ns

ns: not significant

**P ≤ 0.05; **P ≤ 0.01; ***P ≤ 0.001*

Supplementary Table 2. Spearman correlation analysis of the relationship between total (L2-peptide ELISA) and neutralizing (PBNA) antibody levels, listed according to each M8mer antigen group and tested HPV type.

HPV type	Spearman Analysis	M8mer V1	M8mer V2	M8mer V3	M8mer V4	M8mer V5	M8mer V6
**HPV16**	p value	0.1323	0.9349	0.5821	0.2675	0.7793	0.0368
	Spearman r	0.5952	-0.0476	-0.2381	0.4524	0.1198	0.7619
	p value summary	ns	ns	ns	ns	ns	*
**HPV18**	p value	0.0218	0.0154	0.3268	0.3268	0.0694	0.1966
	Spearman r	0.8095	0.8333	0.4048	0.4048	0.6905	0.5238
	p value summary	*	*	ns	ns	ns	ns
**HPV31**	p value	0.2992	0.171	0.0576	0.4844	0.5821	0.2431
	Spearman r	0.4286	0.5476	-0.7143	0.2874	0.2381	0.4762
	p value summary	ns	ns	ns	ns	ns	ns
**HPV33**	p value	0.1966	0.0218	0.8401	0.0072	0.3894	0.0576
	Spearman r	0.5238	0.8095	-0.0952	0.881	0.3571	0.7143
	p value summary	ns	*	ns	**	ns	ns

ns: not significant

**P ⩽ 0.05; **P ⩽ 0.01; ***P ⩽ 0.001*

Supplementary Table 3. Properties of the monoclonal antibodies (mAbs) utilized in this study.

mAb	isotype	target epitope[1]	L2-peptide ELISA[2]	PBNA[3]	Cross-neutralizing[4]
1MK2L2	IgG1	HPV1 L2 aa16-35	pos	pos	HPV2, HPV3 pos
2TK14L2	IgG1	HPV2 L2 aa18-37	pos	pos	HPV3 pos
3MK1L2	IgG1	HPV3 L2 aa18-37	pos	pos	HPV1, HPV2 pos
4SA1L2	IgG1	HPV4 L2 aa17-35	pos	pos	HPV95 pos
3SA1AL2	IgG1	HPV3 L2 aa18-37	pos	neg	HPV39 pos
3SA1BL2	IgG1	HPV3 L2 aa18-37	pos	neg	HPV39 pos
3SA2L2	IgG1	HPV3 L2 aa18-37	pos	neg	HPV39 pos

1: Epitope homologs to the neutralization epitope in HPV16 L2 (aa20-38).
2: L2-peptide ELISA was performed using the target HPV L2 peptide.
3: PBNA was performed using the targeted HPV.
4: Cross-neutralizing ability as tested by PBNA for other HPV types.
Pos: positive
Neg: negative
