# Supplementary material for: Inter-epitope spacer variation within polytopic L2-based human papillomavirus antigens affects immunogenicity
Source: NPJ Vaccines. 2024 Feb 24;9:44. doi: 10.1038/s41541-024-00832-0 (PMC10894200; doi:10.1038/s41541-024-00832-0)
Supplement: Supplementary file 2 — REPORTING SUMMARY [file 41541_2024_832_MOESM2_ESM.pdf]

nature portfolio

Corresponding author(s): Prof. Dr. Martin Müller

Last updated by author(s): Nov 29, 2023

Reporting Summary

Nature Portfolio wishes to improve the reproducibility of the work that we publish. This form provides structure for consistency and transparency in reporting. For further information on Nature Portfolio policies, see our Editorial Policies and the Editorial Policy Checklist.

Statistics

For all statistical analyses, confirm that the following items are present in the figure legend, table legend, main text, or Methods section.

n/a	Confirmed	
☐	☒	The exact sample size (*n*) for each experimental group/condition, given as a discrete number and unit of measurement
☐	☒	A statement on whether measurements were taken from distinct samples or whether the same sample was measured repeatedly
☐	☒	The statistical test(s) used AND whether they are one- or two-sided<br>*Only common tests should be described solely by name; describe more complex techniques in the Methods section.*
☐	☒	A description of all covariates tested
☐	☒	A description of any assumptions or corrections, such as tests of normality and adjustment for multiple comparisons
☐	☒	A full description of the statistical parameters including central tendency (e.g. means) or other basic estimates (e.g. regression coefficient) AND variation (e.g. standard deviation) or associated estimates of uncertainty (e.g. confidence intervals)
☐	☒	For null hypothesis testing, the test statistic (e.g. *F*, *t*, *r*) with confidence intervals, effect sizes, degrees of freedom and *P* value noted<br>*Give P values as exact values whenever suitable.*
☒	☐	For Bayesian analysis, information on the choice of priors and Markov chain Monte Carlo settings
☐	☒	For hierarchical and complex designs, identification of the appropriate level for tests and full reporting of outcomes
☐	☒	Estimates of effect sizes (e.g. Cohen's *d*, Pearson's *r*), indicating how they were calculated

Our web collection on statistics for biologists contains articles on many of the points above.

Software and code

Policy information about availability of computer code

Data collection	In the submmitted manuscript, authors haven't collected any data from online source. All data were generated in lab by our own.
Data analysis	GraphPad Prism 9 was used for all the statistical calculation. AlphaFold and the online SWISS-MODEL server were utilized for structure prediction.

For manuscripts utilizing custom algorithms or software that are central to the research but not yet described in published literature, software must be made available to editors and reviewers. We strongly encourage code deposition in a community repository (e.g. GitHub). See the Nature Portfolio guidelines for submitting code & software for further information.

Data

Policy information about availability of data

All manuscripts must include a data availability statement. This statement should provide the following information, where applicable:

- Accession codes, unique identifiers, or web links for publicly available datasets
- A description of any restrictions on data availability
- For clinical datasets or third party data, please ensure that the statement adheres to our policy

The important and necessary data were all shown in the manuscript.

Research involving human participants, their data, or biological material

Policy information about studies with human participants or human data. See also policy information about sex, gender (identity/presentation), and sexual orientation and race, ethnicity and racism.

Reporting on sex and gender	N/A
Reporting on race, ethnicity, or other socially relevant groupings	N/A
Population characteristics	N/A
Recruitment	N/A
Ethics oversight	N/A

Note that full information on the approval of the study protocol must also be provided in the manuscript.

Field-specific reporting

Please select the one below that is the best fit for your research. If you are not sure, read the appropriate sections before making your selection.

☒ Life sciences ☐ Behavioural & social sciences ☐ Ecological, evolutionary & environmental sciences

For a reference copy of the document with all sections, see nature.com/documents/nr-reporting-summary-flat.pdf

Life sciences study design

All studies must disclose on these points even when the disclosure is negative.

Sample size	The sample size of eight in our study is sufficient to yield a statistically significant p-value, for a two-tailed Mann-Whitney test, with a study power of 80% and the effect size of 1.6.
Data exclusions	No data were excluded.
Replication	To ensure the reproducibility of the data in our study, we have incorporated at least one replication group. When necessary, a third-time repeating will be conducted.
Randomization	Our animals were randomized grouped. And all treatments on them were comparable among groups.
Blinding	Blinding is not applicable in our study. Because we have to inject groups of mice with different antigens. And because all the data collected from mice were from objective experimental assays.

Reporting for specific materials, systems and methods

We require information from authors about some types of materials, experimental systems and methods used in many studies. Here, indicate whether each material, system or method listed is relevant to your study. If you are not sure if a list item applies to your research, read the appropriate section before selecting a response.

Materials & experimental systems

n/a	Involved in the study	
☐	☒	Antibodies
☐	☒	Eukaryotic cell lines
☒	☐	Palaeontology and archaeology
☐	☒	Animals and other organisms
☒	☐	Clinical data
☒	☐	Dual use research of concern
☒	☐	Plants

Methods

n/a	Involved in the study	
☒	☐	ChIP-seq
☒	☐	Flow cytometry
☒	☐	MRI-based neuroimaging

Antibodies

Antibodies used	The secondary antibody utilized is goat anti-mouse IgG, HRP, from ThermoFisher.

Validation	All the monoclonal antibodies were generated in lab, by co-authors of this manuscript.

Eukaryotic cell lines

Policy information about cell lines and Sex and Gender in Research

Cell line source(s)	Hela cells, LOVO T cells, HEK293 TT cells and hybridoma cells were involved in our study.
Authentication	Authentication by DKFZ.
Mycoplasma contamination	No suspicious Mycoplasma contamination is observed.
Commonly misidentified lines (See ICLAC register)	N/A

Animals and other research organisms

Policy information about studies involving animals; ARRIVE guidelines recommended for reporting animal research, and Sex and Gender in Research

Laboratory animals	6- to 8-week old BALB/c female mice were enrolled.
Wild animals	No wild animals were used in our study.
Reporting on sex	No sex issue should be addressed here.
Field-collected samples	Our study didn't involve samples collected from the field.
Ethics oversight	Animal Ethics Committee: Regional Council of Karlsruhe, Germany; 35–9185.81/G-248/16.

Note that full information on the approval of the study protocol must also be provided in the manuscript.

Plants

Seed stocks	N/A
Novel plant genotypes	N/A
Authentication	N/A
